# Dealing with skin reactions to gloves during the COVID-19 pandemic

**DOI:** 10.1017/ice.2020.212

**Published:** 2020-05-08

**Authors:** Mohammadreza Tabary, Farnaz Araghi, Soheila Nasiri, Sahar Dadkhahfar

**Affiliations:** 1 School of Medicine, Tehran University of Medical Sciences, Tehran, Iran; 2 Skin Research Center, Shahid Beheshti University of Medical Sciences, Tehran, Iran; 3 Department of Dermatology, Loghman Hakim Hospital, Shahid Beheshti University of Medical Sciences, Tehran, Iran

*To the Editor*—Healthcare workers (HCWs) are encouraged to wear gloves by the WHO in the direct care of the patients during the COVID-19 pandemic. Medical gloves are made of different polymers, including latex, nitrile rubber, polyvinyl chloride, polyurethane, and neoprene. Nitrile and latex gloves are preferred during the COVID-19 pandemic due to better durability.^1^ Latex gloves are flexible, fit well, are sensitive to touch, and provide moderate protection.^2^ Vinyl gloves provide moderate protection, are sensitive to touch, but are not as durable.^2^ However, nitrile gloves are chemical- and puncture-resistant and provide the highest level of protection and durability.^2^ Many adverse skin reactions, including irritant contact dermatitis, allergic contact dermatitis, and contact urticaria have been reported with the use of all types of gloves.^3^

Latex gloves are frequently used among HCWs. Hypersensitivity to natural rubber latex (NRL) has been increasingly reported, with an incidence of 2.8% to 17% among HCWs.^3^ HCWs are highly at risk of developing allergic reactions to NRL, especially operating room personnel, dental assistants, laboratory personnel, hospital housekeeping personnel, and ambulance attendants.^4^ Atopic background, history of hand dermatitis, allergy to certain foods, female gender, and multiple exposures are among the risk factors for developing hypersensitivity to NRL.^3^ Hypersensitivity reaction to bananas, avocados, chestnuts, kiwis, and other fruits have been reported among these patients.^5^ Skin reactions include localized pruritus, burning, stinging, contact and generalized urticaria. The most frequently observed reaction is irritant contact dermatitis presenting as dry, crusted, fissuring patches.^4^ In suspected patients, a thorough history of allergic reactions to balloons, gloves, barium enema, and other latex devices should be taken. The gold standard in the diagnosis is skin-prick testing in patients with localized symptoms and latex-specific IgE antibody assessment in cases of systemic symptoms.^5^ However, the wear and/or use test and the patch test are the alternative diagnostic tests.^4^ The most effective approach for the management of latex allergy is personal and environmental avoidance by considering hypoallergic gloves.^4^

**Fig. 1.**
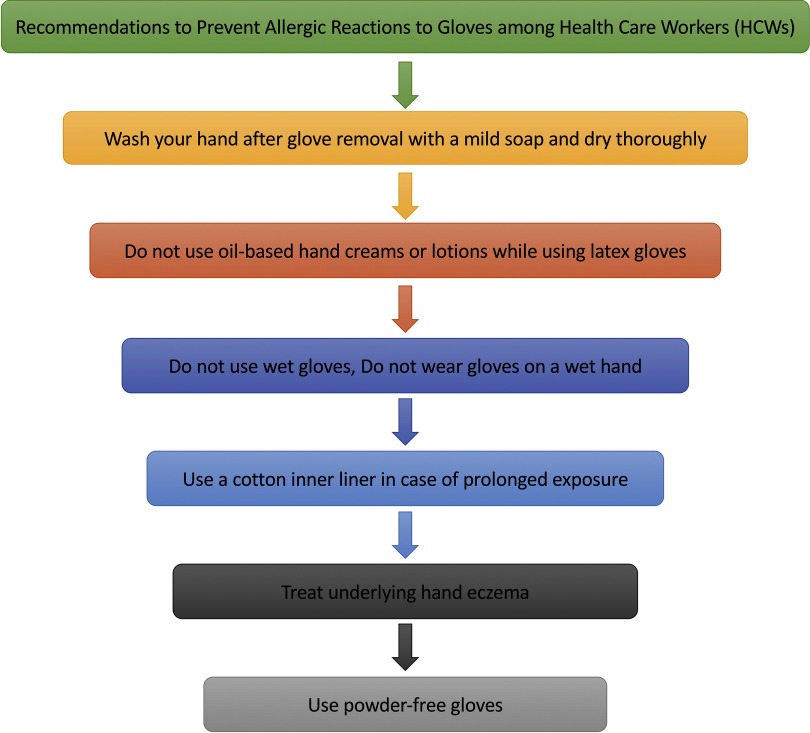
Recommendations to prevent allergic reactions to gloves among healthcare workers.

Recommendations for the prevention of allergic reactions to gloves are summarized in Fig. 1. Urticaria can be treated with antihistamines and the elimination of the antigen. H_1,2_ Blockers can be used before coming into contact with latex devices; however, latex avoidance is superior to this protocol.^4^

Plastic gloves, considered as hypoallergenic polyvinylchloride (PVC) gloves, are also used among HCWs. Contact allergic reaction to PVC has also been reported as a result of allergy to many additives used in these gloves, including carba mix, mercaptobenzothiazole (MBT), thiuram mix, mixed dialkyl thioureas, and black rubber mix.^5^ Allergic contact dermatitis has been reported in numerous case reports. Lesions may also become generalized in some patients. A patch test can be used to confirm the diagnosis. Topical corticosteroids are considered as the best choice of treatment; however, patients should be advised to use other types of gloves,^5^ although allergic contact dermatitis may coexist with immediate hypersensitivity to Latex.^5^

Nitrile, neoprene, and polyurethane are also used in plastic gloves. Hand dermatitis has been reported with these types of materials. The patch test is recommended in suspicious cases. Application of topical and oral steroids can mitigate the symptoms but the benefits should be weighed against the risks of side effects.^6^

Glove-related hand urticaria should also be considered as a differential diagnosis; it is caused by dermographism upon the application of the glove. Pain, burning, and pruritus in the affected area, and systemic symptoms such as fever are not present in glove-related hand urticaria. Further, nitrile gloves are more likely to cause this phenomenon because they are rigid and less flexible.^7^

Some types of powder used in gloves have been associated with an increased risk of skin roughness due to altering glove pH.^8^ Glove powder has been reported to cause allergic reactions, and hand eczema has been reported to decrease significantly after using powder-free gloves.^9^ Thus, the use of powder-free gloves is recommended in the current pandemic situation. HCWs are also encouraged to wear double gloves when handling COVID-19 patients’ airways, blood, urine, and other body fluids. The outer glove should be the first equipment to be removed.^2^
